# Introduction. The neurobiology of drug addiction: new vistas

**DOI:** 10.1098/rstb.2008.0108

**Published:** 2008-07-24

**Authors:** T.W Robbins, B.J Everitt, D.J Nutt

**Affiliations:** 1 Experimental Psychology and Behavioural and Clinical Neuroscience Institute, University of CambridgeCambridge CB2 3EB, UK; 2 Psychopharmacology Unit, University of BristolBristol BS8 1TH, UK

This Royal Society Discussion Meeting, held on 25–26 February 2008, was intended to mark the enormous scientific progress that has been made in this field in the last decade or so, advances that can be measured from the time of a Frontiers Meeting of the Wellcome Trust (Altman *et al*. 1996). At that time, it was already clear that the nucleus accumbens in the ventral striatum of the basal forebrain was a key structure mediating some of the positive reinforcing (‘rewarding’) effects of several drugs of abuse, especially the psychomotor stimulant drugs, including cocaine and amphetamine. The innervation of the nucleus accumbens by the chemical neurotransmitter dopamine was also known to be a key mediator of some of these actions. However, although it was suspected that the nucleus accumbens was merely one node in a complex circuitry at the ‘limbic–striatal interface’, the full nature and functions of this circuitry were still unclear.

In parallel with these developments were new ideas emanating from the realization that certain forms of associative learning, including both Pavlovian and instrumental conditioning, depended upon elements of this system, for natural rewards such as food and sex, as well as for drugs of abuse. It was already clear that relapse derived in part from classical conditioning. Some theorists had begun to speculate that certain aspects of associative conditioning such as stimulus-response habit learning were particularly relevant to understanding drug abuse (e.g. White 1996), but the modern theory of habit learning itself was still being developed in a way that was to prove crucial for understanding the functions of the striatum as a whole. Dopamine had previously been related to ‘reward’ functions (see Wise 2004), but the shortcomings of this bold, but simplistic, notion were already becoming apparent, especially in relation to understanding how, for example, opiate drugs exerted their addictive actions. The concept of ‘opponent motivational systems’, originally developed as a notable psychological theory, was just beginning to be related to opponent neural systems, although the precise neural correlates of the ‘negative’ component were still not well understood. In addition to these basic issues of neural mediation, the question of why only certain individuals become addicted, even after exposure to drugs of abuse, was only just being formulated, and the consequences of drug addiction, including cognitive impairment and possibly lack of self-control, were also mainly a matter for speculation.

Many of these issues have been addressed in the first part of the Discussion Meeting, ‘Theories of Drug Addiction’. Koob & Le Moal (2005), following on from their synthesis, elaborate on the development of their neural opponent motivational theory, which they relate closely to the concepts of homeostasis and allostasis from stress research, and which appears especially relevant to the understanding of opiate abuse. Central to this theory is the concept that withdrawal leads to an aversive motivational state and that much drug-seeking behaviour is directed towards allieviating this state. Koob & Le Moal (2008) also subscribe to a staging of addiction that includes an initial ‘impulsivity’ phase followed by ‘compulsivity’, which forms the central theme of the paper by Everitt *et al*. (2008). These authors demonstrate how impulsive trait-like behaviour leads to compulsive-like cocaine dependence, paralleling a ‘shift’ in the locus of neural control from the ventral to the dorsal striatum, and embracing a possible behavioural transition in instrumental control to dominance by stimulus–response habit-like representations. Robinson & Berridge (2008) counterpoint these two positions via their own ‘incentive salience’ hypothesis with its focus on the sensitization by drugs of abuse of the mesolimbic dopamine system. Finally, Stewart (2008) reviews the considerable advances made in delineating the distinct neural systems underlying relapse, following exposure to stress, drug-related cues or the drug itself.

Much of the advance in understanding of the neurobiology of drug abuse has come from the study of psychomotor stimulant and opiate drugs, but other forms of addiction have been recognized, notably in the case of nicotine, and now, more controversially, in the form of the ‘behavioural addictions’ of gambling and compulsive eating. The questions now being asked are whether there are similar neural mechanisms underlying these extensions to the concept of addiction. Hence, in the section entitled ‘Extending the Concept of Addiction’, Markou (2008) illustrates the use of applying similar concepts and methods to the understanding of nicotine addiction, as have been used for the psychomotor stimulants. In particular, the advent of drugs with glutamatergic or GABAergic actions is shown to have implications for the treatment of nicotine dependence. This is paralleled by Stephens & Duka's (2008) article on neural mechanisms underlying alcohol dependence, which shows how binge drinking in humans can be modelled in rodents to suggest important changes in glutamatergic mediated excitability and reductions in neuronal plasticity (long-term potentiation) in limbic structures such as the amygdala and hippocampus. Potenza (2008) surveys the burgeoning information concerning compulsive gambling. Much of this concerns human behaviour and has emerged from the use of brain imaging techniques that have revealed a remarkable commonality of neural systems mediating the reinforcing properties of drugs and money. He also considers the comorbidity of gambling with substance dependence (e.g. especially alcoholism) and where similar issues of trait impulsivity are seen as contributing to individual vulnerability to gambling behaviour. Finally, the Director of the US National Institute on Drug Abuse, Nora Volkow, updates and extends her classic analysis (e.g. Volkow *et al*. 2003) of neurochemical changes present in substance abusers (using positron emission tomography, PET) to include obese individuals and animal models of obesity. These studies also raise the issue of the possibly causal role of baseline individual differences in striatal dopamine receptor binding that are also taken up in the contributions by Everitt *et al*. (2008) and Nader *et al*. (2008; see below). She also introduced an idea that has emerged from both the animal (e.g. Jentsch & Taylor 1999; Robbins & Everitt 1999) and the human literature (Rogers & Robbins 2001) that substance abuse can arise from an impairment of top-down inhibitory control, presumably arising from impairments in frontal lobe function.

The next group of papers, ‘Vulnerability to drug abuse’, appropriately consider genetic and environmental factors contributing to the neurobiology of drug abuse, especially in the vanguard of the Human Genome Project. Crabbe (2008) focuses on the contribution of behavioural genetics, mainly in the mouse, to the understanding of alcoholism, where there is perhaps the best evidence of heritability. He also considers the likely importance of epigenetic factors. Wong & Schumann (2008) introduce strategies addressing the heterogeneity and polygenicity of substance use based on the identification of more homogeneous subgroups of patients and the characterization of genes contributing to their phenotype via linkage and association studies. They also advocate functional genetic analysis based on endophenotypes and animal behavioural experimentation. By contrast, in work parallel to that of Volkow *et al*. (2008), Nader *et al*. (2008) present the results of an exceptionally systematic series of PET studies of rhesus monkeys before and after their exposure to cocaine. The crucial findings were of reductions in striatal D_2_ dopamine receptor binding being found *before* drug exposure, implying that this change was not simply a consequence of drug abuse, but may be predisposing to it. Everitt *et al*. (2008) had also described the recent similar findings of Dalley *et al*. (2007) for the rat, where reductions in D_2_ dopamine receptor binding had been associated with enhanced impulsivity. Of course, the question arises about the origin of such predisposing changes, whether, for example, they depend on specific genes or are the product of environmental factors. In the case of the non-human primates, Nader *et al*. (2008) stress the fact that the low dopamine receptor binding is associated with likely stress resulting from social neglect. Shaham and colleagues, in the paper by Crombag *et al*. (2008), return to the environmental factors through which individuals relapse to substance abuse, specifically according to contextual conditioning. This article thus complements the contribution by Stewart (2008) in defining the neural substrates of contextual reinstatement of drug-seeking behaviour.

The final section considers ‘Causes and Consequences of Addiction’. Nestler (2008) begins by reviewing the considerable advances over the last decade in understanding of the intracellular molecular changes associated with chronic exposure to drugs of abuse, in particular biochemical changes in proteins such as the transcription factor ΔFosB and its gene targets. He is approaching this question by the use of DNA expression arrays coupled with the analysis of chromatin remodelling—changes in the posttranslational modifications of histones at drug-regulated gene promoters. His findings establish chromatin remodelling as an important regulatory mechanism underlying drug-induced behavioural plasticity, and promise to reveal fundamentally new insight into how ΔFosB contributes to addiction by regulating the expression of specific target genes in brain reward pathways. The paper by Porrino (Beveridge *et al*. 2008) surveys the consequences of chronic drug exposure through self-administration in monkeys that provide further evidence for ramifying effects beyond the initial sites of drug action in the ventral striatum to the dorsal striatum and cortex, established by the methods including autoradiography and the measurement of cerebral metabolism in the temporal and frontal lobes. These neuroanatomical changes are also accompanied by impairments in cognitive function such as visual recognition memory, which are also demonstrated to occur in human drug abusers, using comparable neuropsychological tests. Garavan *et al*. (2008) also consider the sequelae of drug addiction in human substance abusers from the vantage of functional brain imaging using magnetic resonance. They provide direct evidence that inhibitory control is impaired in cocaine abusers, associated with altered activity of the prefrontal cortex during performance of the stop signal reaction time task. Moreover, they show surprisingly that intravenous cocaine actually *improves* performance in this task, not only in behavioural terms but also by normalizing brain activity in lateral and medial regions of the prefrontal cortex. These findings controversially suggest that one possible factor contributing to the susceptibility to stimulant addiction is a drive to self-medication. The last contribution by O'Brien (2008) focused specifically on treatment of addiction and surveyed the large number of strategies that are currently in vogue, particularly from a pharmaceutical viewpoint. New treatments have emerged over the past decade, many based on the advances derived from basic neurobiology, and there are obvious indications for opiate (e.g. buprenorphine), nicotine (e.g. patches) and alcohol (e.g. acamprosate, naltrexone) abuse, as well as more speculative candidate treatments such as vaccination. However, drug companies still needed encouragement to innovate in this area, and any treatment for cocaine dependence is still problematic.

The discussion provoked by the meeting was a measure of the contemporary interest in this field from the general public, as well as the scientific community. Some of the current excitement has been engendered in the UK by a Technology Foresight Initiative in *Brain Science, Addiction and Drugs*, published as *Drugs and the Future: Brain Science, Addiction and Society* (Nutt *et al*. 2007). This initiative has recently been the subject of a similarly named *Academy of Medical Sciences* publication, containing recommendations for the UK government for future policy in the field of drug addiction and related fields, such as the treatment by drugs of mental illness and cognitive enhancing drugs. The present Discussion Meeting has helped to highlight the vibrancy of this field in the UK and internationally.
